# *N*-[(3*RS*,4*RS*)-1-Benzyl-4-methyl­piperidin-3-yl]-5-nitro-1-phenyl­sulfonyl-1*H*-pyrrolo­[2,3-*b*]pyridine-4-amine

**DOI:** 10.1107/S1600536812039979

**Published:** 2012-09-29

**Authors:** Ellen Pfaffenrot, Dieter Schollmeyer, Stefan Laufer

**Affiliations:** a Eberhard-Karls-University Tübingen, Auf der Morgenstelle 8, 72076 Tübingen, Germany; b Institute of Organic Chemistry, University Mainz, Duesbergweg 10-14, 55099 Mainz, Germany

## Abstract

The pyrrolo­pyridine system in the title compound, C_27_H_29_N_5_O_4_S, is oriented at a dihedral angle of 71.20 (5)° towards the phenyl ring of the tosyl residue and at a dihedral angle of 45.43 (4)° towards the benzyl group. The structure shows an intra­molecular N—H⋯O and a weak intra­molecular N—H⋯N hydrogen bond. The piperidine ring adopts a chair conformation, with the *cis* substituents displaying a torsion angle of −54.59 (18)°.

## Related literature

For inhibitors of Janus kinases, see: Hoffmann-La Roche AG (2011 ▶).
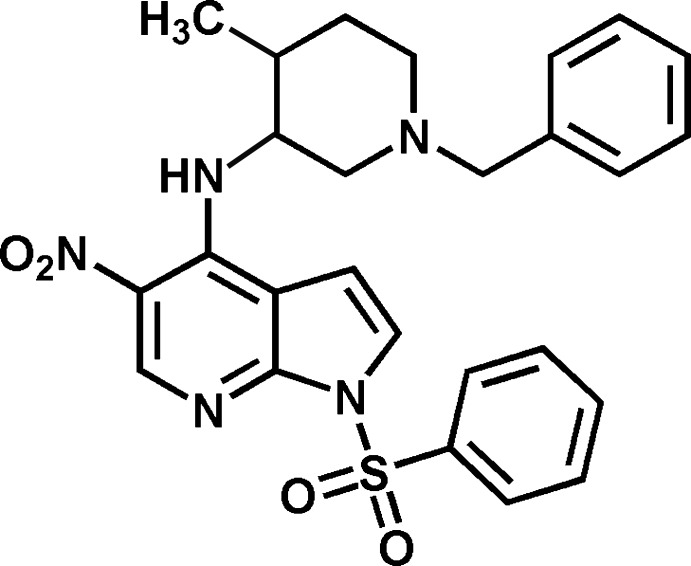


## Experimental

### 

#### Crystal data

C_27_H_29_N_5_O_4_S
*M* *_r_* = 519.61
Monoclinic, 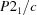

*a* = 17.2626 (10) Å
*b* = 11.5411 (8) Å
*c* = 13.1313 (9) Å
β = 98.443 (3)°
*V* = 2587.8 (3) Å^3^
*Z* = 4
Mo *K*α radiation
μ = 0.17 mm^−1^
*T* = 173 K
0.50 × 0.20 × 0.08 mm

#### Data collection

Bruker APEXII diffractometer
25499 measured reflections
6140 independent reflections
4286 reflections with *I* > 2σ(*I*)
*R*_int_ = 0.035

#### Refinement

*R*[*F* ^2^ > 2σ(*F* ^2^)] = 0.039
*wR*(*F* ^2^) = 0.117
*S* = 1.02
6140 reflections
336 parameters
H-atom parameters constrained
Δρ_max_ = 0.25 e Å^−3^
Δρ_min_ = −0.35 e Å^−3^

### 

Data collection: *APEX2* (Bruker, 2006 ▶); cell refinement: *APEX2*; data reduction: *APEX2*; program(s) used to solve structure: *SIR97* (Altomare *et al.*, 1999 ▶); program(s) used to refine structure: *SHELXL97* (Sheldrick, 2008 ▶); molecular graphics: *PLATON* (Spek, 2009 ▶); software used to prepare material for publication: *PLATON*.

## Supplementary Material

Crystal structure: contains datablock(s) I, global. DOI: 10.1107/S1600536812039979/bt6841sup1.cif

Structure factors: contains datablock(s) I. DOI: 10.1107/S1600536812039979/bt6841Isup2.hkl

Supplementary material file. DOI: 10.1107/S1600536812039979/bt6841Isup3.cml

Additional supplementary materials:  crystallographic information; 3D view; checkCIF report

## Figures and Tables

**Table 1:** Hydrogen-bond geometry (Å, °)

*D*—H⋯*A*	*D*—H	H⋯*A*	*D*⋯*A*	*D*—H⋯*A*
N23—H23⋯O21	0.92	1.95	2.6322 (17)	130
N23—H23⋯N26	0.92	2.35	2.8235 (18)	111

**Fig. 1.**
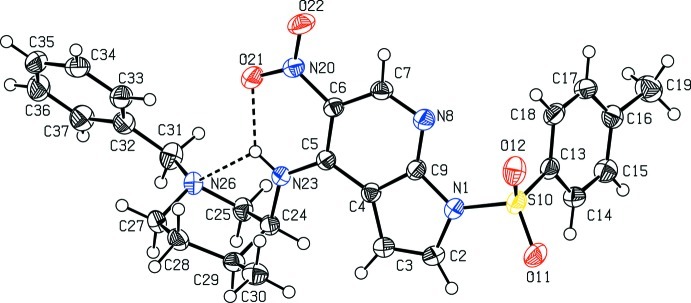
Crystal structure of the title compound with labelling and displacement ellipsoids drawn at the 50% probability level. Hydrogen bonds are represented as dashed lines.

## supplementary crystallographic information

### Comment

*N*-(1-benzyl-4-methylpiperidin-3-yl)-5-nitro-1-tosyl-1*H*- pyrrolo[2,3-*b*]pyridine-4-amine is an important intermediate in the synthesis of tricyclic heterocyclic compounds being inhibitors of Janus kinases (Hoffmann-La Roche AG, 2011).

The tosyl residue is oriented in a dihedral angle of 71.2 (0)° towards the pyrrolo-pyridine system and shows a dihedral angle of 45.4 (0)° to the benzyl group (Fig. 1). The methylene group in the title compound presents an angle of 112.0 (5)° between the phenyl and piperidine residue. The equatorial methyl and the axial pyrrolo-pyridine substituent of the piperidine show a torsion angle of 54.5 (9)°. The intramolecular hydrogen bonds N23—H23···O21 1.95 Å and N23—H23···N26 2.35 Å stabilize the molecular conformation (Tabl. 1).

### Experimental

The compound was prepared by nucleophilic substitution of 4-chloro-5-nitro-1-tosyl-1*H*-pyrrolo[2,3-*b*]pyridine with *cis*-1-benzyl-3-aminopiperidine in the presence of tertiary amine base. A mixture of 4-chloro-5-nitro-1-tosyl-1*H*-pyrrolo[2,3-*b*]pyridine (0.624 g, 1.778 mmol), *cis*-1-benzyl-3-aminopiperidine-dihydrobromide (0.781 g, 2.133 mmol) and diisopropylethylamine (1.9 ml, 10.666 mmol) in 2-propanol (3 ml) was heated in a microwave reactor at 353 K for 1 h. The reaction media was concentrated under vacuum and the product precipitated from methanol to yield 0.855 g (92%) of the title compound. Crystals were obtained by recrystallization in methanol at 277 K.

### Refinement

All hydrogen atom were located in a difference Fourier map. Nevertheless, they were refined using a riding model with N—H = 0.92 Å, C—H = 0.95 Å (aromatic) or 0.98–0.99 Å (*sp*^3^ C-atom). The isotropic displacement parameters were set to 1.2–1.5 times of the *U*_eq_ of the parent atom.

### Crystal data

**Table:**

C_27_H_29_N_5_O_4_S	*F*(000) = 1096
*M**_r_* = 519.61	*D*_x_ = 1.334 Mg m^−^^3^
Monoclinic, *P*2_1_/*c*	Mo *K*α radiation, λ = 0.71073 Å
Hall symbol: -P 2ybc	Cell parameters from 5909 reflections
*a* = 17.2626 (10) Å	θ = 2.4–27.4°
*b* = 11.5411 (8) Å	µ = 0.17 mm^−^^1^
*c* = 13.1313 (9) Å	*T* = 173 K
β = 98.443 (3)°	Plate, yellow
*V* = 2587.8 (3) Å^3^	0.50 × 0.20 × 0.08 mm
*Z* = 4	

### Data collection

**Table:**

Bruker APEXII diffractometer	4286 reflections with *I* > 2σ(*I*)
Radiation source: sealed Tube	*R*_int_ = 0.035
Graphite monochromator	θ_max_ = 27.9°, θ_min_ = 2.1°
CCD scan	*h* = −22→22
25499 measured reflections	*k* = −12→15
6140 independent reflections	*l* = −17→17

### Refinement

**Table:**

Refinement on *F*^2^	Primary atom site location: structure-invariant direct methods
Least-squares matrix: full	Secondary atom site location: difference Fourier map
*R*[*F*^2^ > 2σ(*F*^2^)] = 0.039	Hydrogen site location: inferred from neighbouring sites
*wR*(*F*^2^) = 0.117	H-atom parameters constrained
*S* = 1.02	*w* = 1/[σ^2^(*F*_o_^2^) + (0.0633*P*)^2^ + 0.2813*P*] where *P* = (*F*_o_^2^ + 2*F*_c_^2^)/3
6140 reflections	(Δ/σ)_max_ < 0.001
336 parameters	Δρ_max_ = 0.25 e Å^−^^3^
0 restraints	Δρ_min_ = −0.35 e Å^−^^3^

### Special details

**Table:**

Geometry. All e.s.d.'s (except the e.s.d. in the dihedral angle between two l.s. planes) are estimated using the full covariance matrix. The cell e.s.d.'s are taken into account individually in the estimation of e.s.d.'s in distances, angles and torsion angles; correlations between e.s.d.'s in cell parameters are only used when they are defined by crystal symmetry. An approximate (isotropic) treatment of cell e.s.d.'s is used for estimating e.s.d.'s involving l.s. planes.
Refinement. Refinement of *F*^2^ against ALL reflections. The weighted *R*-factor *wR* and goodness of fit *S* are based on *F*^2^, conventional *R*-factors *R* are based on *F*, with *F* set to zero for negative *F*^2^. The threshold expression of *F*^2^ > σ(*F*^2^) is used only for calculating *R*-factors(gt) *etc*. and is not relevant to the choice of reflections for refinement. *R*-factors based on *F*^2^ are statistically about twice as large as those based on *F*, and *R*- factors based on ALL data will be even larger.

### Fractional atomic coordinates and isotropic or equivalent isotropic displacement parameters (Å^2^)

**Table:**

	*x*	*y*	*z*	*U*_iso_*/*U*_eq_	
N1	0.25756 (7)	0.38269 (12)	0.13550 (10)	0.0321 (3)	
C2	0.30403 (10)	0.38599 (15)	0.05701 (12)	0.0341 (4)	
H2	0.2851	0.3892	−0.0146	0.041*	
C3	0.37997 (10)	0.38382 (15)	0.09919 (12)	0.0328 (4)	
H3	0.4234	0.3859	0.0624	0.039*	
C4	0.38439 (9)	0.37788 (13)	0.20968 (11)	0.0261 (3)	
C5	0.44591 (8)	0.37411 (12)	0.29571 (11)	0.0249 (3)	
C6	0.41701 (9)	0.37441 (13)	0.39271 (11)	0.0257 (3)	
C7	0.33663 (9)	0.37230 (14)	0.39967 (12)	0.0303 (3)	
H7	0.3220	0.3714	0.4666	0.036*	
N8	0.27971 (8)	0.37148 (12)	0.32056 (10)	0.0339 (3)	
C9	0.30663 (9)	0.37682 (13)	0.22942 (12)	0.0279 (3)	
S10	0.15864 (2)	0.37973 (4)	0.11393 (3)	0.03343 (12)	
O11	0.13997 (7)	0.40111 (11)	0.00604 (9)	0.0432 (3)	
O12	0.13179 (7)	0.45534 (11)	0.18685 (10)	0.0425 (3)	
C13	0.13327 (9)	0.23692 (14)	0.13895 (12)	0.0302 (3)	
C14	0.12459 (9)	0.15711 (16)	0.05869 (13)	0.0347 (4)	
H14	0.1380	0.1774	−0.0066	0.042*	
C15	0.09609 (10)	0.04762 (15)	0.07540 (13)	0.0362 (4)	
H15	0.0898	−0.0073	0.0209	0.043*	
C16	0.07654 (9)	0.01692 (15)	0.17065 (13)	0.0341 (4)	
C17	0.08870 (10)	0.09693 (16)	0.25082 (13)	0.0369 (4)	
H17	0.0773	0.0757	0.3169	0.044*	
C18	0.11701 (9)	0.20656 (16)	0.23604 (13)	0.0350 (4)	
H18	0.1253	0.2604	0.2913	0.042*	
C19	0.04180 (12)	−0.10047 (17)	0.18642 (16)	0.0490 (5)	
H19A	0.0185	−0.1323	0.1197	0.073*	
H19B	0.0013	−0.0927	0.2310	0.073*	
H19C	0.0830	−0.1526	0.2188	0.073*	
N20	0.46852 (8)	0.37004 (11)	0.48969 (10)	0.0286 (3)	
O21	0.54003 (7)	0.35745 (12)	0.49078 (9)	0.0436 (3)	
O22	0.44069 (7)	0.37755 (12)	0.57041 (9)	0.0450 (3)	
N23	0.52257 (7)	0.37061 (11)	0.28858 (10)	0.0283 (3)	
H23	0.5578	0.3611	0.3478	0.034*	
C24	0.56077 (9)	0.35130 (14)	0.19766 (12)	0.0283 (3)	
H24	0.5213	0.3224	0.1399	0.034*	
C25	0.62288 (9)	0.25754 (14)	0.22722 (13)	0.0318 (4)	
H25A	0.5979	0.1867	0.2498	0.038*	
H25B	0.6486	0.2377	0.1669	0.038*	
N26	0.68097 (7)	0.30109 (11)	0.31072 (10)	0.0298 (3)	
C27	0.72550 (9)	0.39528 (15)	0.27122 (13)	0.0344 (4)	
H27A	0.7483	0.3682	0.2105	0.041*	
H27B	0.7688	0.4198	0.3248	0.041*	
C28	0.67087 (9)	0.49632 (15)	0.24132 (13)	0.0344 (4)	
H28A	0.7001	0.5584	0.2115	0.041*	
H28B	0.6528	0.5276	0.3040	0.041*	
C29	0.59923 (9)	0.46310 (14)	0.16334 (12)	0.0304 (3)	
H29	0.6182	0.4459	0.0966	0.036*	
C30	0.54279 (11)	0.56531 (15)	0.14510 (14)	0.0387 (4)	
H30A	0.5207	0.5815	0.2082	0.058*	
H30B	0.5004	0.5463	0.0894	0.058*	
H30C	0.5710	0.6337	0.1259	0.058*	
C31	0.73136 (10)	0.20900 (15)	0.36030 (14)	0.0388 (4)	
H31A	0.7643	0.1782	0.3108	0.047*	
H31B	0.6983	0.1449	0.3795	0.047*	
C32	0.78383 (9)	0.25168 (15)	0.45576 (13)	0.0334 (4)	
C33	0.75798 (10)	0.33474 (16)	0.51998 (13)	0.0364 (4)	
H33	0.7063	0.3645	0.5044	0.044*	
C34	0.80684 (11)	0.37471 (16)	0.60677 (13)	0.0378 (4)	
H34	0.7887	0.4323	0.6492	0.045*	
C35	0.88179 (10)	0.33065 (16)	0.63118 (14)	0.0409 (4)	
H35	0.9150	0.3570	0.6908	0.049*	
C36	0.90805 (10)	0.24790 (17)	0.56794 (15)	0.0446 (5)	
H36	0.9595	0.2174	0.5843	0.054*	
C37	0.85963 (10)	0.20925 (16)	0.48089 (15)	0.0401 (4)	
H37	0.8785	0.1530	0.4378	0.048*	

### Atomic displacement parameters (Å^2^)

**Table:**

	*U*^11^	*U*^22^	*U*^33^	*U*^12^	*U*^13^	*U*^23^
N1	0.0277 (7)	0.0408 (8)	0.0267 (7)	−0.0019 (6)	−0.0003 (6)	0.0009 (6)
C2	0.0370 (9)	0.0408 (10)	0.0240 (8)	−0.0038 (7)	0.0026 (7)	0.0002 (7)
C3	0.0321 (8)	0.0401 (10)	0.0266 (8)	−0.0028 (7)	0.0053 (7)	−0.0004 (7)
C4	0.0281 (8)	0.0253 (8)	0.0246 (8)	−0.0015 (6)	0.0029 (6)	−0.0010 (6)
C5	0.0271 (8)	0.0212 (7)	0.0261 (8)	−0.0015 (6)	0.0031 (6)	−0.0003 (6)
C6	0.0292 (8)	0.0237 (8)	0.0236 (8)	0.0009 (6)	0.0023 (6)	0.0002 (6)
C7	0.0322 (8)	0.0351 (9)	0.0244 (8)	0.0023 (6)	0.0068 (6)	0.0003 (6)
N8	0.0293 (7)	0.0435 (9)	0.0291 (7)	0.0015 (6)	0.0044 (6)	0.0005 (6)
C9	0.0277 (8)	0.0290 (8)	0.0257 (8)	−0.0007 (6)	−0.0002 (6)	0.0005 (6)
S10	0.0277 (2)	0.0367 (2)	0.0334 (2)	0.00088 (16)	−0.00356 (16)	0.00128 (17)
O11	0.0400 (7)	0.0491 (8)	0.0362 (7)	−0.0019 (5)	−0.0088 (5)	0.0102 (6)
O12	0.0333 (6)	0.0407 (7)	0.0520 (8)	0.0052 (5)	0.0013 (6)	−0.0075 (6)
C13	0.0230 (7)	0.0383 (9)	0.0280 (8)	0.0023 (6)	−0.0000 (6)	−0.0009 (7)
C14	0.0335 (9)	0.0442 (10)	0.0269 (8)	−0.0011 (7)	0.0059 (7)	−0.0008 (7)
C15	0.0361 (9)	0.0387 (10)	0.0341 (9)	0.0031 (7)	0.0061 (7)	−0.0058 (7)
C16	0.0255 (8)	0.0384 (10)	0.0382 (10)	0.0057 (7)	0.0037 (7)	0.0053 (7)
C17	0.0329 (9)	0.0509 (11)	0.0271 (9)	0.0018 (7)	0.0044 (7)	0.0052 (7)
C18	0.0310 (8)	0.0471 (10)	0.0257 (8)	0.0011 (7)	0.0002 (7)	−0.0051 (7)
C19	0.0497 (11)	0.0448 (12)	0.0529 (12)	−0.0017 (9)	0.0094 (9)	0.0082 (9)
N20	0.0339 (7)	0.0263 (7)	0.0251 (7)	−0.0004 (5)	0.0027 (6)	0.0007 (5)
O21	0.0289 (6)	0.0701 (9)	0.0303 (6)	0.0010 (6)	−0.0004 (5)	0.0047 (6)
O22	0.0461 (7)	0.0661 (9)	0.0230 (6)	0.0075 (6)	0.0057 (5)	−0.0026 (6)
N23	0.0244 (6)	0.0367 (8)	0.0235 (7)	−0.0032 (5)	0.0028 (5)	−0.0005 (5)
C24	0.0263 (8)	0.0338 (9)	0.0254 (8)	−0.0031 (6)	0.0054 (6)	−0.0048 (6)
C25	0.0286 (8)	0.0313 (9)	0.0355 (9)	−0.0028 (6)	0.0048 (7)	−0.0061 (7)
N26	0.0254 (6)	0.0303 (7)	0.0331 (7)	−0.0020 (5)	0.0024 (6)	−0.0021 (6)
C27	0.0266 (8)	0.0424 (10)	0.0349 (9)	−0.0085 (7)	0.0066 (7)	−0.0012 (7)
C28	0.0357 (9)	0.0336 (9)	0.0350 (9)	−0.0102 (7)	0.0086 (7)	−0.0011 (7)
C29	0.0332 (8)	0.0337 (9)	0.0252 (8)	−0.0025 (7)	0.0072 (7)	−0.0010 (6)
C30	0.0468 (10)	0.0344 (10)	0.0359 (10)	0.0006 (8)	0.0095 (8)	0.0027 (7)
C31	0.0342 (9)	0.0367 (10)	0.0440 (10)	0.0054 (7)	0.0014 (8)	−0.0058 (8)
C32	0.0311 (8)	0.0334 (9)	0.0358 (9)	0.0017 (7)	0.0051 (7)	0.0041 (7)
C33	0.0315 (8)	0.0428 (10)	0.0347 (9)	0.0042 (7)	0.0043 (7)	0.0028 (8)
C34	0.0443 (10)	0.0403 (10)	0.0295 (9)	−0.0015 (8)	0.0078 (7)	0.0027 (7)
C35	0.0402 (10)	0.0455 (11)	0.0341 (9)	−0.0083 (8)	−0.0037 (8)	0.0096 (8)
C36	0.0310 (9)	0.0505 (12)	0.0500 (11)	0.0037 (8)	−0.0014 (8)	0.0118 (9)
C37	0.0358 (9)	0.0386 (10)	0.0457 (11)	0.0069 (7)	0.0049 (8)	0.0036 (8)

### Geometric parameters (Å, º)

**Table:**

N1—C9	1.3916 (19)	N23—C24	1.4633 (19)
N1—C2	1.396 (2)	N23—H23	0.9205
N1—S10	1.6899 (13)	C24—C25	1.533 (2)
C2—C3	1.346 (2)	C24—C29	1.548 (2)
C2—H2	0.9500	C24—H24	1.0000
C3—C4	1.443 (2)	C25—N26	1.462 (2)
C3—H3	0.9500	C25—H25A	0.9900
C4—C9	1.404 (2)	C25—H25B	0.9900
C4—C5	1.433 (2)	N26—C31	1.464 (2)
C5—N23	1.3409 (19)	N26—C27	1.469 (2)
C5—C6	1.435 (2)	C27—C28	1.515 (2)
C6—C7	1.404 (2)	C27—H27A	0.9900
C6—N20	1.4431 (19)	C27—H27B	0.9900
C7—N8	1.321 (2)	C28—C29	1.534 (2)
C7—H7	0.9500	C28—H28A	0.9900
N8—C9	1.347 (2)	C28—H28B	0.9900
S10—O12	1.4224 (13)	C29—C30	1.526 (2)
S10—O11	1.4276 (12)	C29—H29	1.0000
S10—C13	1.7488 (17)	C30—H30A	0.9800
C13—C18	1.390 (2)	C30—H30B	0.9800
C13—C14	1.391 (2)	C30—H30C	0.9800
C14—C15	1.385 (2)	C31—C32	1.517 (2)
C14—H14	0.9500	C31—H31A	0.9900
C15—C16	1.388 (2)	C31—H31B	0.9900
C15—H15	0.9500	C32—C37	1.391 (2)
C16—C17	1.393 (2)	C32—C33	1.393 (2)
C16—C19	1.508 (3)	C33—C34	1.393 (2)
C17—C18	1.380 (2)	C33—H33	0.9500
C17—H17	0.9500	C34—C35	1.383 (3)
C18—H18	0.9500	C34—H34	0.9500
C19—H19A	0.9800	C35—C36	1.385 (3)
C19—H19B	0.9800	C35—H35	0.9500
C19—H19C	0.9800	C36—C37	1.387 (3)
N20—O22	1.2294 (17)	C36—H36	0.9500
N20—O21	1.2409 (17)	C37—H37	0.9500
			
C9—N1—C2	108.33 (13)	N23—C24—C29	111.99 (13)
C9—N1—S10	128.04 (11)	C25—C24—C29	110.39 (13)
C2—N1—S10	123.56 (11)	N23—C24—H24	109.3
C3—C2—N1	109.03 (14)	C25—C24—H24	109.3
C3—C2—H2	125.5	C29—C24—H24	109.3
N1—C2—H2	125.5	N26—C25—C24	108.94 (13)
C2—C3—C4	108.60 (14)	N26—C25—H25A	109.9
C2—C3—H3	125.7	C24—C25—H25A	109.9
C4—C3—H3	125.7	N26—C25—H25B	109.9
C9—C4—C5	118.18 (13)	C24—C25—H25B	109.9
C9—C4—C3	105.97 (13)	H25A—C25—H25B	108.3
C5—C4—C3	135.85 (14)	C25—N26—C31	112.41 (13)
N23—C5—C4	124.80 (14)	C25—N26—C27	109.05 (13)
N23—C5—C6	122.51 (13)	C31—N26—C27	112.75 (13)
C4—C5—C6	112.69 (13)	N26—C27—C28	108.85 (13)
C7—C6—C5	122.25 (14)	N26—C27—H27A	109.9
C7—C6—N20	115.39 (13)	C28—C27—H27A	109.9
C5—C6—N20	122.27 (13)	N26—C27—H27B	109.9
N8—C7—C6	125.24 (15)	C28—C27—H27B	109.9
N8—C7—H7	117.4	H27A—C27—H27B	108.3
C6—C7—H7	117.4	C27—C28—C29	113.01 (14)
C7—N8—C9	112.61 (13)	C27—C28—H28A	109.0
N8—C9—N1	123.02 (14)	C29—C28—H28A	109.0
N8—C9—C4	128.92 (14)	C27—C28—H28B	109.0
N1—C9—C4	108.06 (13)	C29—C28—H28B	109.0
O12—S10—O11	120.90 (8)	H28A—C28—H28B	107.8
O12—S10—N1	107.21 (7)	C30—C29—C28	110.19 (14)
O11—S10—N1	103.78 (7)	C30—C29—C24	113.50 (13)
O12—S10—C13	109.66 (8)	C28—C29—C24	110.62 (13)
O11—S10—C13	108.85 (8)	C30—C29—H29	107.4
N1—S10—C13	105.21 (7)	C28—C29—H29	107.4
C18—C13—C14	120.95 (16)	C24—C29—H29	107.4
C18—C13—S10	119.98 (13)	C29—C30—H30A	109.5
C14—C13—S10	118.93 (13)	C29—C30—H30B	109.5
C15—C14—C13	119.02 (16)	H30A—C30—H30B	109.5
C15—C14—H14	120.5	C29—C30—H30C	109.5
C13—C14—H14	120.5	H30A—C30—H30C	109.5
C14—C15—C16	120.97 (16)	H30B—C30—H30C	109.5
C14—C15—H15	119.5	N26—C31—C32	112.05 (14)
C16—C15—H15	119.5	N26—C31—H31A	109.2
C15—C16—C17	118.82 (16)	C32—C31—H31A	109.2
C15—C16—C19	120.47 (16)	N26—C31—H31B	109.2
C17—C16—C19	120.71 (16)	C32—C31—H31B	109.2
C18—C17—C16	121.24 (16)	H31A—C31—H31B	107.9
C18—C17—H17	119.4	C37—C32—C33	118.20 (16)
C16—C17—H17	119.4	C37—C32—C31	120.57 (16)
C17—C18—C13	118.91 (16)	C33—C32—C31	121.22 (14)
C17—C18—H18	120.5	C32—C33—C34	120.92 (16)
C13—C18—H18	120.5	C32—C33—H33	119.5
C16—C19—H19A	109.5	C34—C33—H33	119.5
C16—C19—H19B	109.5	C35—C34—C33	120.08 (17)
H19A—C19—H19B	109.5	C35—C34—H34	120.0
C16—C19—H19C	109.5	C33—C34—H34	120.0
H19A—C19—H19C	109.5	C34—C35—C36	119.47 (17)
H19B—C19—H19C	109.5	C34—C35—H35	120.3
O22—N20—O21	120.82 (13)	C36—C35—H35	120.3
O22—N20—C6	119.35 (13)	C35—C36—C37	120.35 (16)
O21—N20—C6	119.82 (13)	C35—C36—H36	119.8
C5—N23—C24	128.99 (13)	C37—C36—H36	119.8
C5—N23—H23	118.8	C36—C37—C32	120.97 (18)
C24—N23—H23	110.5	C36—C37—H37	119.5
N23—C24—C25	106.45 (13)	C32—C37—H37	119.5
			
C9—N1—C2—C3	−0.75 (19)	C14—C15—C16—C17	−2.3 (2)
S10—N1—C2—C3	−177.98 (12)	C14—C15—C16—C19	176.94 (16)
N1—C2—C3—C4	0.49 (19)	C15—C16—C17—C18	2.3 (2)
C2—C3—C4—C9	−0.05 (18)	C19—C16—C17—C18	−176.92 (16)
C2—C3—C4—C5	−179.48 (17)	C16—C17—C18—C13	0.2 (2)
C9—C4—C5—N23	177.61 (14)	C14—C13—C18—C17	−2.8 (2)
C3—C4—C5—N23	−3.0 (3)	S10—C13—C18—C17	172.83 (12)
C9—C4—C5—C6	−2.37 (19)	C7—C6—N20—O22	−7.8 (2)
C3—C4—C5—C6	177.00 (17)	C5—C6—N20—O22	175.66 (14)
N23—C5—C6—C7	−176.77 (14)	C7—C6—N20—O21	171.34 (14)
C4—C5—C6—C7	3.2 (2)	C5—C6—N20—O21	−5.2 (2)
N23—C5—C6—N20	−0.5 (2)	C4—C5—N23—C24	−10.8 (2)
C4—C5—C6—N20	179.48 (13)	C6—C5—N23—C24	169.18 (14)
C5—C6—C7—N8	−1.0 (2)	C5—N23—C24—C25	−130.72 (15)
N20—C6—C7—N8	−177.51 (14)	C5—N23—C24—C29	108.55 (17)
C6—C7—N8—C9	−2.1 (2)	N23—C24—C25—N26	−62.82 (15)
C7—N8—C9—N1	−176.84 (14)	C29—C24—C25—N26	58.93 (17)
C7—N8—C9—C4	3.0 (2)	C24—C25—N26—C31	166.21 (13)
C2—N1—C9—N8	−179.41 (15)	C24—C25—N26—C27	−67.99 (16)
S10—N1—C9—N8	−2.3 (2)	C25—N26—C27—C28	65.74 (16)
C2—N1—C9—C4	0.71 (17)	C31—N26—C27—C28	−168.66 (13)
S10—N1—C9—C4	177.77 (11)	N26—C27—C28—C29	−55.95 (18)
C5—C4—C9—N8	−0.7 (2)	C27—C28—C29—C30	174.23 (14)
C3—C4—C9—N8	179.72 (16)	C27—C28—C29—C24	47.91 (18)
C5—C4—C9—N1	179.14 (13)	N23—C24—C29—C30	−54.59 (18)
C3—C4—C9—N1	−0.40 (17)	C25—C24—C29—C30	−173.00 (13)
C9—N1—S10—O12	44.89 (16)	N23—C24—C29—C28	69.86 (16)
C2—N1—S10—O12	−138.45 (14)	C25—C24—C29—C28	−48.55 (17)
C9—N1—S10—O11	173.91 (13)	C25—N26—C31—C32	−172.15 (14)
C2—N1—S10—O11	−9.43 (16)	C27—N26—C31—C32	64.09 (18)
C9—N1—S10—C13	−71.80 (15)	N26—C31—C32—C37	−144.17 (16)
C2—N1—S10—C13	104.86 (14)	N26—C31—C32—C33	35.3 (2)
O12—S10—C13—C18	−21.20 (15)	C37—C32—C33—C34	0.3 (3)
O11—S10—C13—C18	−155.49 (12)	C31—C32—C33—C34	−179.11 (16)
N1—S10—C13—C18	93.81 (13)	C32—C33—C34—C35	−1.0 (3)
O12—S10—C13—C14	154.50 (12)	C33—C34—C35—C36	0.9 (3)
O11—S10—C13—C14	20.22 (15)	C34—C35—C36—C37	−0.1 (3)
N1—S10—C13—C14	−90.49 (13)	C35—C36—C37—C32	−0.6 (3)
C18—C13—C14—C15	2.8 (2)	C33—C32—C37—C36	0.5 (3)
S10—C13—C14—C15	−172.86 (12)	C31—C32—C37—C36	179.90 (16)
C13—C14—C15—C16	−0.2 (2)		

### Hydrogen-bond geometry (Å, º)

**Table:**

*D*—H···*A*	*D*—H	H···*A*	*D*···*A*	*D*—H···*A*
N23—H23···O21	0.92	1.95	2.6322 (17)	130
N23—H23···N26	0.92	2.35	2.8235 (18)	111
